# Proteomic dynamics in endochondral ossification: insights from antler tip analysis

**DOI:** 10.7717/peerj.21568

**Published:** 2026-07-27

**Authors:** Xi Xi, Xinyue Cao, Yuying Zhou, Zichen Tian, Nanqi Hou, Zuoyang Li, Zhenwei Zhou, Xiangyan Li, Hang Su

**Affiliations:** 1 Northeast Asia Research Institute of Traditional Chinese Medicine, Changchun University of Chinese Medicine, Changchun, Jilin, China; 2 School of Pharmaceutical Sciences, Changchun University of Chinese Medicine, Changchun, Jilin, China

**Keywords:** Antler regeneration, Proteomics, Endochondral ossification, Proteome dynamics, Cluster analysis

## Abstract

**Background:**

The antler primary growth center, located at the distal tip of the growing antler, comprises five consecutive tissue zones beneath the velvet skin. Because the outermost reserve mesenchyme contains blastema progenitor cells with multipotent differentiation capacity, antler regeneration recapitulates embryonic skeletal development through endochondral ossification (ECO). The molecular mechanisms governing cell fate decisions and tissue morphogenesis across these zones remain poorly understood.

**Methods:**

We profiled the proteome of the sika deer (*Cervus nippon*) antler growth center across all five tissue zones using the Orbitrap Astral platform in data-independent acquisition (DIA) mode. Protein identification and quantification were performed with DIA-NN. Proteome dynamics were analyzed by integrating three complementary clustering approaches including weighted gene co-expression network analysis (WGCNA), Fuzzy C-means clustering, and monotonic feature selection. Differentially expressed proteins were annotated by gene ontology (GO) enrichment analysis. Transcriptomic data from the same tissue zones were reanalyzed to assess mRNA–protein concordance.

**Results:**

A total of 8,173 proteins were identified across the five tissue zones, representing the most comprehensive proteomic coverage of the antler growth center. Clustering analyses resolved distinct protein expression modules corresponding to sequential ECO stages from mesenchymal proliferation in the reserve mesenchyme to cartilage mineralization in the innermost zone. Deep proteome coverage enabled detection of low-abundance chondrogenic transcription factors SOX9, SOX6, and RUNX3, whose spatial expression matched their known roles in chondrogenesis. At the protein level, the reserve mesenchyme co-expressed 11 embryonic and 23 mesenchymal stem cell markers, indicating that antler stem cells represented a unique mesenchymal stem cell (MSC) population possessing partial embryonic stem cell (ESC)-like features.

**Conclusions:**

This study provides a spatially resolved proteomic atlas of the complete antler growth center and identifies key regulatory proteins at each stage of endochondral ossification. The findings offer new insights into progenitor cell characteristics, transcription factor activity, and protein dynamics during bone development, with potential relevance to bone repair and regenerative medicine.

## Introduction

Deer (*Cervidae*), among ungulates, possess a distinctive secondary sexual characteristic known as antlers. The antlers undergo complete regeneration annually, setting them apart from all the other known secondary sexual traits. They are the only mammalian appendages capable of full regeneration, growing at an astonishing rate of more than two cm per day during their fast antler growth stage (Li, Yang & Sheppard, 2009). The tip of the growing antler, known as the antler growth center (ATC), provided an exceptional *in vivo* model to study the entire spectrum of endochondral ossification (ECO) in a spatially organized manner (Ba et al., 2019). The ATC is histologically organized into five consecutive tissue zones that reflect a process of ECO. The zones consist of: (1) a reserve mesenchyme (RM) zone, abundant in progenitor cells; (2) a precartilage (PC) zone, in which cells differentiate towards the chondrogenic lineage (Hu et al., 2024); (3) a transition zone (TZ) (Zhang et al., 2018); (4) a cartilage (CA) zone, marked by mature chondrocytes and significant extracellular matrix production; and (5) a mineralized cartilage (MC) zone, where chondrocytes undergo hypertrophy and the matrix undergoes calcification. This spatial organization of the ATC provides an opportunity to capture molecular “snapshots” of each distinct stage of bone development, from mesenchymal cell proliferation through differentiation to terminal ossification.

To understand the molecular mechanisms underlying intricate biological processes such as chondrogenesis, high-throughput ‘omics’ technologies have been established (To et al., 2024). An RNA-seq study of ATC identified thousands of genes that changed across the five zones and highlighted the Wnt signaling pathway as a key driver of cell proliferation and differentiation (Ba et al., 2019). However, mRNA abundance does not always reflect protein abundance due to post-transcriptional regulation (Harnik et al., 2021), and proteins are the direct regulators of biological process. This motivated the application of proteomics to the antler model. The first proteome map of red deer (*Cervus elaphus*) antlers revealed the substantial differences between the growing tip and the proximal bony tissue (Park et al., 2004). Subsequent quantitative proteomic studies identified HIF-1 and PI3K-AKT as crucial signaling pathways of antler regeneration by comparing potentiated and dormant pedicle periosteal cells (Dong et al., 2016). A label-free quantitative proteomic study analyzed the antler tip at six different developmental stage, identifying 875 differentially expressed proteins (DEPs) and revealing an up-regulation of chondrogenesis-associated proteins during the growth period and lysosome-related proteins during the ossification stages (Zhang, Li & Xing, 2021a). An integrative transcriptomic and proteomic analysis of red deer (*Cervus elaphus*) antler cartilage further implicated Wnt signaling in rapid antler growth (Chen et al., 2022). Another comprehensive study employed both RNA-seq and proteomics to examine gene and protein expression differences across four distinct tissue zones of the *Elaphurus davidianus* (milu deer) antler tip (RM, PC, TZ, CA) (Liu et al., 2024). This work identified 4,611 DEGs and 2388 DEPs, emphasizing the roles of collagen, integrin, and solute carrier families in antler tissue remodeling. It indicated that antler growth mechanisms might be similar to cancer cell proliferation.

Despite this progress, three major gaps remained. Firstly, none of the existing integrated multi-omics studies profiled all five tissue zones, including the mineralized cartilage zone. The final stage of bone formation is thus still uncharacterized at the protein level. Secondly, transcription factors have never been detected in previous antler proteomics study. This is because conventional proteomic platforms cannot detect proteins with low abundance. Thirdly, the extent of divergence between protein and mRNA levels throughout the bone development process has never been systematically measured. The Orbitrap Astral platform, with deeper proteome coverage than conventional Orbitrap instruments, allows us to address all three of these gaps in current study. Deep proteomics based on Astral platform can achieve deeper proteome coverage than previous studies, enabling the identification of low-abundance regulatory proteins including transcriptional factors that are crucial for antler regeneration during ECO process. Employing three clustering methods, we test the protein variations across different bone development zones and assess the disparity in protein and mRNA expression. These findings provided a proteomic landscape of bone development in a regenerating organ and a resource for the discovery of novel therapeutic targets in bone repair and regenerative medicine.

## Materials & Methods

### Collection of five consecutive tissues from the antler growth center

Antler tissues were collected from three 5-year-old male sika deer (*Cervus nippon*) at 45 days after antler regeneration. The collection details were consistent with previous published protocol (Li et al., 2002). The animal antler sampling method used in this study were reviewed and approved by the Ethics Committee of Changchun University of Chinese Medicine (Approval No. 2024-974). Animals were housed in standard deer paddocks at the Shuang-yang deer farm under natural photoperiod conditions, with ad libitum access to water and standard cervid diet. Antler collection was performed under veterinary supervision following sedation with xylazine hydrochloride (0.1 mg/kg intramuscular injection). The distal six cm of antler tips from main beam was obtained and then sectioned sagittally along the longitudinal axis. Five tissue zones were dissected and then cut into five mm pieces, which were then snap-frozen in liquid nitrogen and stored at −80 °C for further proteomic analysis.

### Sample preparation for proteomics

Protein extraction of antler samples was in accordance with the standard protocol (Wisniewski et al., 2009). Each sample was ground smoothly in liquid nitrogen and then 20 mg of lyophilized sample powder were lysed with 180 µL SDT buffer (containing 100 mM DTT, 4% SDS, 100 mM Tris/HCl, pH 7.6), followed by 10 min of ultrasonication on ice. After incubation for 15 min at 95 °C and ice-bath for 2 min, the lysate was centrifuged at 13,000 g for 10 min at 4 °C. The protein concentration of the supernatant was determined by the BCA method and the supernatant was diluted to 1 µg/µL. 50 µL of the protein sample (50 µg) in SDT buffer was added into 450 µL IAM buffer (50 mM IAM in 8 M urea, pH 8) and incubated for 1 min while shaking at room temperature. Then samples were completely mixed with 4-fold volume of pre-cooled acetone by vortexing and being incubated at −20 °C for 4 h. Samples were then centrifuged at 13,000 g for 15 min at 4 °C and the precipitation was collected. After washing with one mL cold acetone, the pellet was dissolved completely by dissolution buffer (DB buffer, 8 M Urea, 100 mM Tris/HCl, pH 8.5). DB buffer (50 mM NH_4_CO_3_ buffer containing 0.5 µg of Trypsin) was added to each protein sample to a total volume of 100 µL and samples were digested at 37 °C overnight. Formic acid was mixed with the digested sample, adjusted to pH under 3, and centrifuged at 13,000 g for 5 min at room temperature. The supernatant was slowly loaded to the C18 desalting column, washed with washing buffer (0.1% formic acid, 3% acetonitrile) 3 times, and then collected with elution buffer (0.1% formic acid, 70% acetonitrile). The eluents of each sample were collected and lyophilized for LC-MS/MS analysis.

### LC-MS/MS analysis-DIA mode

Astral platform parameters were matched thoses as previously published (Guzman et al., 2024; Heil et al., 2023). Mobile phase A (100% water, 0.1% formic acid) and B (80% acetonitrile, 0.1% formic acid) was prepared separately. The lyophilized powder was dissolved with 10 µL of mobile phase A, centrifuged at 14,000 g for 20 min at 4 °C, and 200 ng of the supernatant was injected for detection. A Thermo Fisher Scientific Vanquish Neo UHPLC system was equipped with a C18 pre-column (5 mm × 300 µm, 5 µm, Thermo Fisher Scientific, Waltham, MA, USA) and a C18 analytical column (PepMap™ Neo UHPLC, 150 µm × 15 cm, 2 µm, Thermo Fisher Scientific, Waltham, MA, USA). The gradient elution was performed at 800 nL/min for 22 min, with mobile phase B increasing from 8% to 55%. Thermo Orbitrap Astral mass spectrometer with an Easy-spray (ESI) ion source was employed. The ion spray voltage was set to 1.9 kV. The ion transfer tube temperature was set to 290 °C, and the mass spectrum was in a data-independent acquisition mode, with a full first-stage mass spectrometry scanning range of m/z 380–980. The MS1 resolution was set to 240,000 (200 m/z) and AGC was set to 500%. The isolation window of parent ion was set to 2 Th and the number of DIA windows was 300. The NCE was set to 25%, the secondary m/z acquisition range was from 150 to 2,000. The MS2 resolution Astral was set to 80,000, and the maximal injection time was 3 ms.

### Identification and quantitation of protein based on DIA-NN

The raw files were searched and analyzed using the DIA-NN library search software (Demichev et al., 2020). The library search parameters were set as follows: a mass tolerance of 10 ppm for precursor ions and 0.02 Da for fragment ions. Cysteine was modified by alkylation, methionine was oxidatively modified, and N-terminal modifications included acetylation, loss of methionine, and loss of methionine with acetylation. A maximum of one missed cleavage site was allowed. To improve the quality of the analytical results, the DIA-NN software further filtered the search results by retaining only credible Peptide Spectrum Matches (PSMs) with a confidence level greater than 99%. Only credible spectral peptides and proteins were retained, and FDR validation was performed to remove peptides and proteins with an FDR greater than 1%. Protein abundances were quantified by the MaxLFQ algorithm and normalized using DIA-NN’s default RT-dependent normalization. The protein intensity matrix exported from DIA-NN was imported into Perseus for downstream processing. Missing values were imputed by random sampling from a normal distribution, which assumes that missing values predominantly reflect proteins below the detection limit. Protein quantitative data were further analyzed by protti R package (Quast, Schuster & Picotti, 2022) to estimate the quality of protein data by assessing the coefficients of variation (CVs) between replicates.

### Fuzzy c-means clustering for protein expression matrix across antler growth tips

The protein expression datasets were clustered by Fuzzy c-means clustering from Mfuzz R package (Kumar & EF, 2007). Prior to clustering, protein expression values were standardized to z-scores across the five tissue zones. The number of clusters (*k* = 10) was determined using the minimum centroid distance criterion implemented in Mfuzz, which identifies the k value at which adding further clusters produces diminishing separation between cluster centroids. The fuzzifier parameter (*m* = 2.0) is the standard default for biological expression data, and reflects a moderate degree of cluster overlap appropriate for continuous developmental gradients. Proteins with a maximum membership score below 0.5 were excluded from cluster-specific analyses to retain only high-confidence assignments. The 51 previously identified antler regeneration marker genes (Ba et al., 2019; Qin et al., 2023) were reassigned to 10 clusters to classify them based on their dynamic changes.

### WGCNA analysis for protein expression matrix across antler growth tips

Protein correlation network analysis was conducted for 15 antler samples using the WGCNA R package (Langfelder & Horvath, 2008). Through utilizing the topological overlap matrix derived from a pairwise relationship-based adjacency matrix, we assessed the neighborhood similarity among proteins. Subsequently, protein co-expression modules, represented by distinct colors, were identified through average linkage hierarchical clustering. Nine modules were successfully identified in total by employing the Dynamic Hybrid Tree Cut algorithm with a minimum module size of 30 proteins.

### Reanalysis of RNA-seq data across the antler tips

The files of reference genome and gene model annotation were directly downloaded from Ensemble genome website (https://ftp.ensembl.org/pub/release-114/fasta/cervus_hanglu_yarkandensis/). Index of the reference genome was built using Hisat2 version 2.2.1 and paired-end clean reads were aligned to the reference genome using Hisat2 (Kim et al., 2019). Samtools was used to sort the resulting SAM files and convert to BAM files. Feature Counts version 2.0.0 (Liao, Smyth & Shi, 2014) was used to count the number of reads mapped to each gene in Cervus Genome. And FPKM of each gene was calculated based on the length of the gene and reads count mapped to this gene. Differential expression analysis of two groups was performed using limma Bioconductor package (Ritchie et al., 2015). Limma provide statistical routines for determining differential expression (fold change ≥ 2) in gene expression matrices using limma-voom approach. The resulting *p*-values were adjusted using the Benjamini and Hochberg’s approach for controlling the false discovery rate. Genes with an adjusted *p*-value ≤ 0.05 found by limma were assigned as differentially expressed genes.

### Identification of monotonically expressed proteins or mRNA

To identify monotonically expressed proteins or mRNA whose expression patterns were highly correlated with ECO process during Antler regeneration, MFSelector (monotonic feature selector) method (Wang et al., 2015) was applied to protein expression or gene expression matrices. The significance of these patterns was evaluated utilizing a permutation test. Two distinct sets of ME (with corresponding *p*-values) were found with ascending or descending monotonic expression patterns for protein or mRNA. To meet the efficient level of stringency for monotonicity, we defined the parameters as permut = 100, svde times = 100, and svde noise = 0.1, according to a previous protocol (Tsai et al., 2022). Finally, total discriminating error value (DEtotal) ≤ 1 were considered as significant monotonically expressed proteins or mRNA with *p*-value ≤ 0.05.

### Correlation between mRNA and protein expression

To correlate mRNA and protein expression between two consecutive stages, we combined proteomic and RNA-seq datasets to obtain 7,993 differentially expression genes or proteins. Correlation analysis of mRNA and protein expression between two consecutive stages was carried out in these 7,993 genes using Pearson’s correlation.

## Results

### In-depth proteomics on deer antler growth center across five consecutive tissues

**Figure 1:**
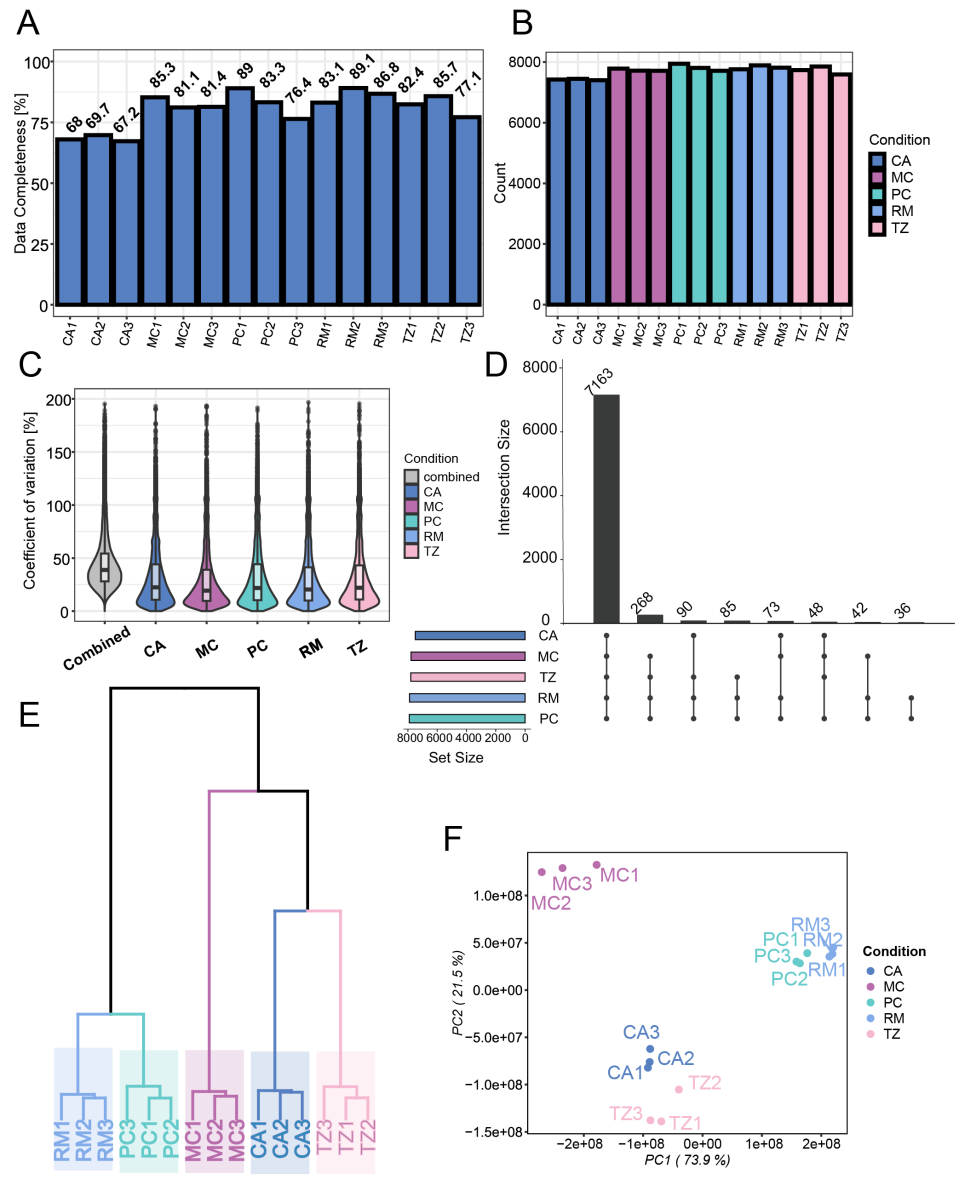
Quality control and overview of the deer antler growth center proteome. (A) Completeness in each of the five tissue zones including reserve mesenchyme (RM), pre-cartilage (PC), transition zone (TZ), cartilage (CA), and mineralized cartilage (MC). (B) The number of identified protein groups across the five tissues. (C) Coefficients of variation between replicates in each tissue group. (D) Upset plot of protein identified across the five tissues. (E) Hierarchical cluster of protein expression in five tissue groups. (F) Principal component analysis (PCA) of protein expression profiles across all 15 samples (5 zones × 3 biological replicates).

Based on orbitrap Astral MS, we comprehensively profiled the dynamic protein expression located in antler growth tip which comprised five distinctive tissue zones including reserve mesenchyme, pre-cartilage, transition zone, cartilage, and mineralized cartilage. For each tissue zone 20 mg of each sample were used for proteomics in biological triplicate. Based on the quality control assessment of proteomics data, it was observed that the completeness of each group ranged from 67% to 89% (Fig. 1A). In order to further assess the reliability of deep proteomics based on orbitrap Astral MS, the number of protein groups in all biological triplicates in each of the five tissue zones was examined. The identification number of replicate samples within certain groups was also highly similar (Fig. 1B). Furthermore, we found that the Pearson’s correlation coefficient for each tissue group is over 0.9, indicating highly consistent measurement (Fig. S1). Notably, the protein identification rate in the cartilage group was lower than the other four groups (Figs. 1A and 1B). In total, 87,402 peptides and 8,173 proteins were identified across the whole antler tip, and the five tissue zones possess 7,163 of common proteins (Fig. 1D; Table S1). The CVs for individual groups range from 19% to 23% (Fig. 1C). The CVs in the “combined” group, which encompassed CVs across all samples rather than the replicates of a certain group, showed that 38.2% of these combined CVs were significantly higher than those of any individual group (Fig. 1C). This observation suggested that protein expression levels exhibited moderate variability across the five tissues. The unsupervised clustering of the protein expression profiles indicated that cartilage and transition zone closely clustered together, and the cluster expanded to mineralized cartilage. Furthermore, reserve mesenchyme and pre-cartilage were further separated in another expanded cluster (Fig. 1E). The identical pattern can be reproduced by principal component analysis (PCA), a distinct clustering method (Fig. 1F). The results revealed sample clustering distance trees that nearly aligned with the experimental sampling scheme significantly. Both dimensionality reduction and clustering methods demonstrated that the cartilage tissue from the transition zone to the cartilage zone already exhibited notable divergence from RM and PC tissues. This suggests that the primary shift in protein expression profiles initiates in the transition zone (TZ).

### Screening antler regeneration related genes from antler proteome atlas

**Figure 2:**
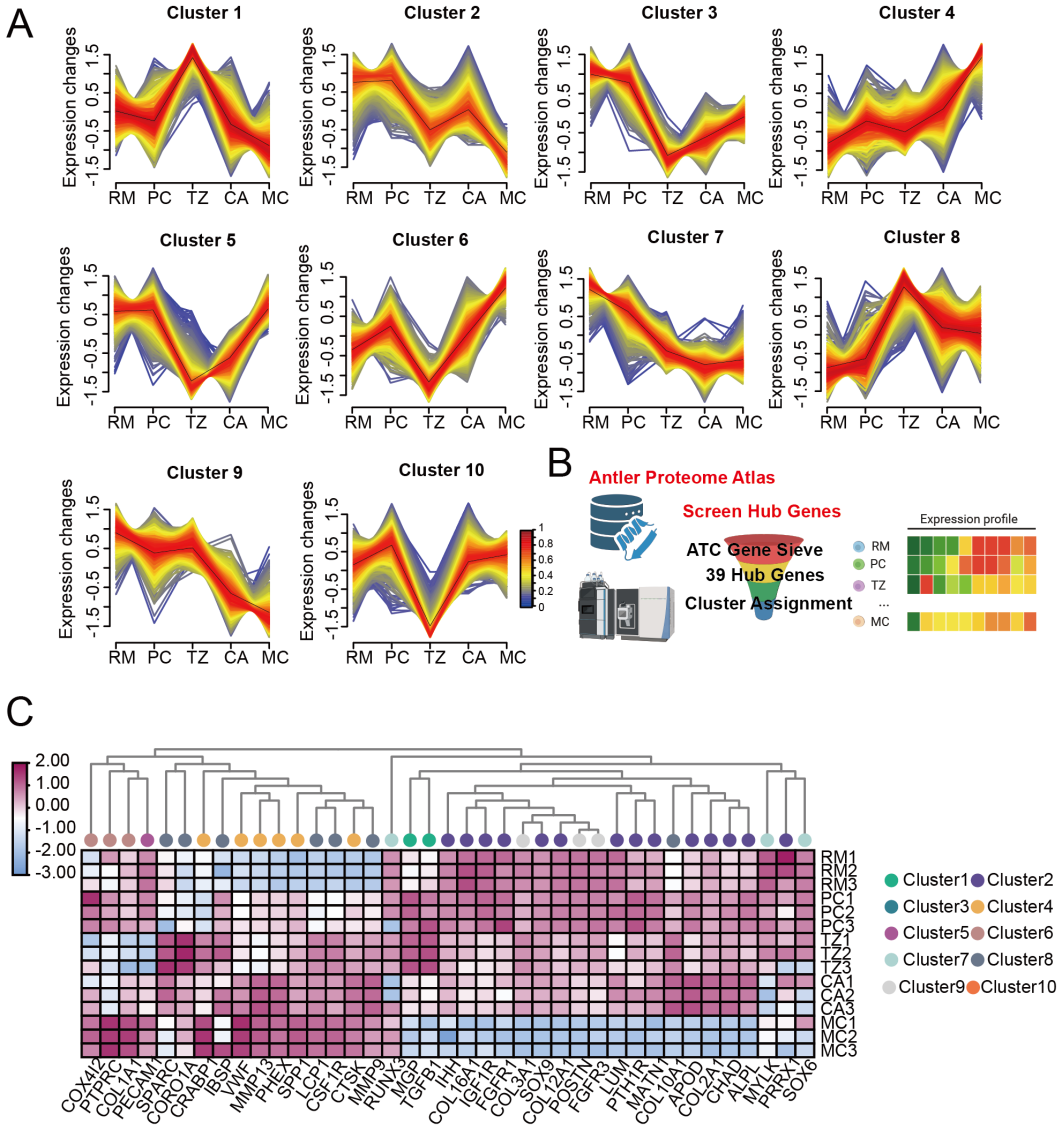
Spatial profiles of protein expression across the antler growth center. (A) Fuzzy c-means clustering identified 10 distinct spatial patterns. *X* axis represent five different tissues, while *y* axis represents log2-transformed, normalized intensity ratios in each group. (B) The workflow of hub gene screening, and 39 hub genes filtered from previously reported 51 key genes involved with antler regeneration to build related protein expression heatmap. (C) Heatmap showing the protein expression abundance of 39 identified antler regeneration hub genes that were assigned to 10 distinct cluster. Figure created using BioRender.

In order to analyze dynamic profile in protein expression, the fuzzy c-means algorithm was applied to group protein expression profiles in five sequential tissue zones of the antler tip. A total of 10 unique clusters were obtained to depict spatial characteristics of proteins that were regulated differently, reflecting variations of protein expression across antler tip (Fig. 2A). Among these, cluster 4 and 8 represented proteins that were upregulated, clusters 2, 7 and 9 represented proteins that were downregulated, whereas clusters 1, 3, 5, 6 and 10 represented proteins displaying a bi-modal expression pattern (Fig. 2A). To better explain the functional module of each identified cluster, we annotated the cluster based on previous published hub genes related with antler regeneration. 29 hub genes closely associated with antler regeneration in antler tips were demonstrated in bulk RNA-seq study (Ba et al., 2019), and 22 new marker genes associated with the classification of antler tip cells were identified from previous single-cell transcriptomic study (Qin et al., 2023). By integrating hub genes associated with antler tip cells and marker genes indicative of antler tip cell classification, we summarized 51 hub genes that were highly relevant to the antler tip growth center, which we termed ATC hub genes as a “antler study sieve” to profile proteomic dynamics across antler tip (Fig. 2B). These genes were subsequently analyzed for their distribution across 10 distinct clusters. In total, we identified 39 ATC proteins, which were predominantly assigned to cluster 2, 4, and 8 (Fig. 2C).

### Bone development marker protein expression dynamics across antler tips

**Figure 3:**
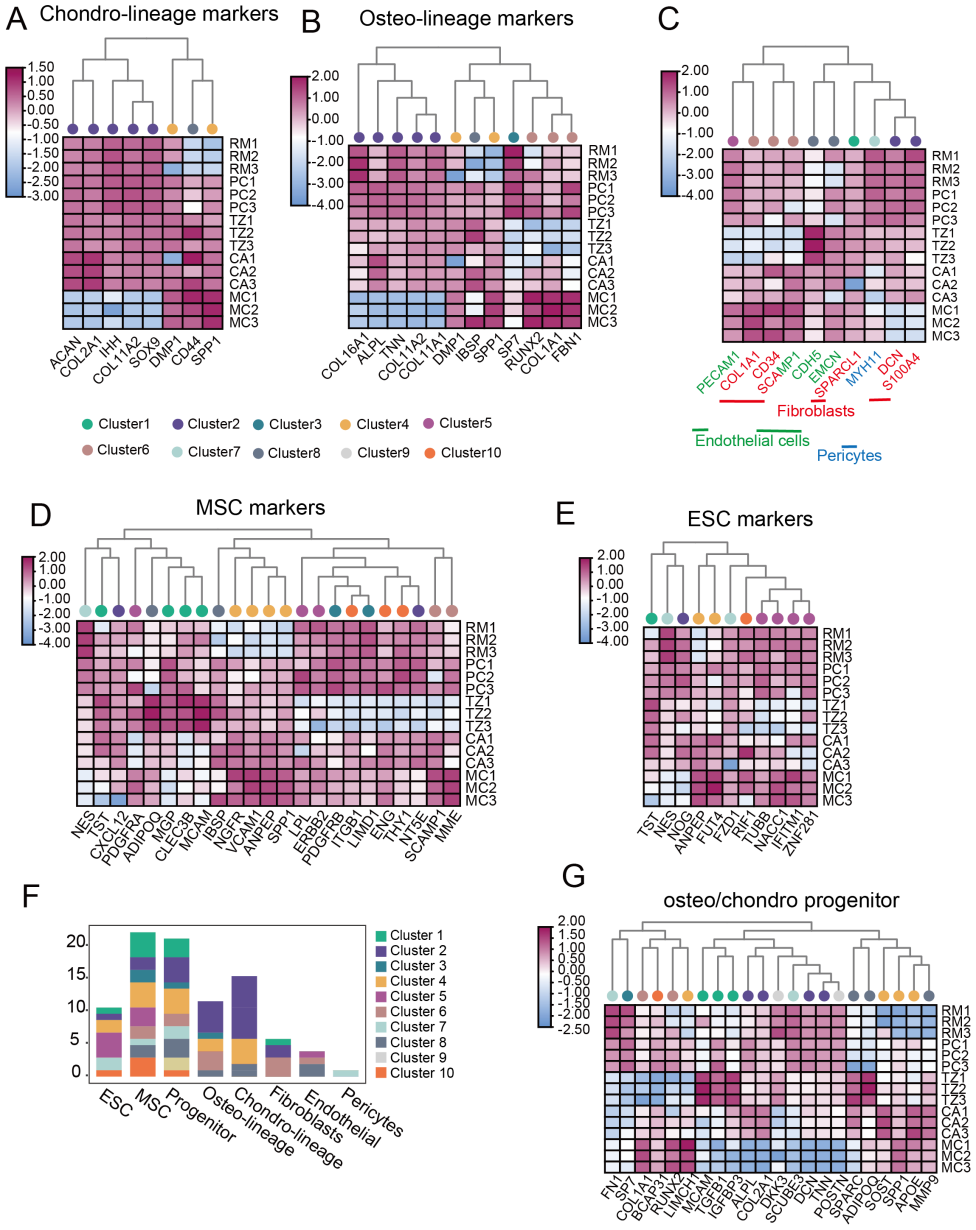
Bone development marker protein expression dynamics across antler tissue zones. (A) Identified chondro-lineage marker expression heatmap from antler proteome. (B) Identified osteo-lineage marker expression heatmap from antler proteome. (C) Identified fibroblasts, endothelial, pericytes marker expression heatmap from antler proteome. (D) Identified mesenchymal stem cell (MSC) marker expression heatmap from antler proteome. (E) Identified embryonic stem cell (ESC) marker expression heatmap from antler proteome. (F) Summary of the number of identified markers classified into 10 clusters. (G) Identified osteo/chondro-progenitor marker expression heatmap from antler proteome.

To explore the key regulatory proteins in antler growth, we integrated 143 previous reported genes specific to mesenchymal stem cells and related non-mesenchymal stem cells involved in bone development as cell class marker genes (Mabuchi et al., 2021; Wang et al., 2019). Among them, a total of 79 proteins were identified to further decipher the heterogeneity of antler tips. The majority of chondro-lineage markers were assigned into cluster 2 (Figs. 3A and 3F). The proteomic results revealed that mineralized cartilage zone (MC) exhibited distinct characteristics compared to the pre-cartilage and cartilage regions, with chondrocyte markers (SOX9, COL2A1, ACAN) expressed at markedly low levels (Fig. 3A). Twelve osteogenic markers were identified and allocated to cluster 6, in addition to cluster 2 (Figs. 3B and 3F). Fibroblast marker genes were predominantly localized in clusters 2 and 6, exhibiting completely opposite expression patterns (Figs. 3C and 3F). In the current study, we identified 22 osteo/chondro progenitor markers from a total of 34 (Fig. 3G). To elucidate the cellular characteristics at the antler growth tip, we firstly analyzed the distribution of all identified proteins within the marker genes of embryonic stem cells (ESC) and mesenchymal stem cells (MSC). We found that the presence of 11 ESC markers out of 50 and 23 MSC markers out of 30 (Figs. 3D and 3E, respectively), suggesting a strong mesenchymal stem cell preference in the antler tip. This is also consistent with previous antler stem cell study findings (Wang et al., 2019).

### WGCNA analysis of protein expression in antler growth center

**Figure 4:**
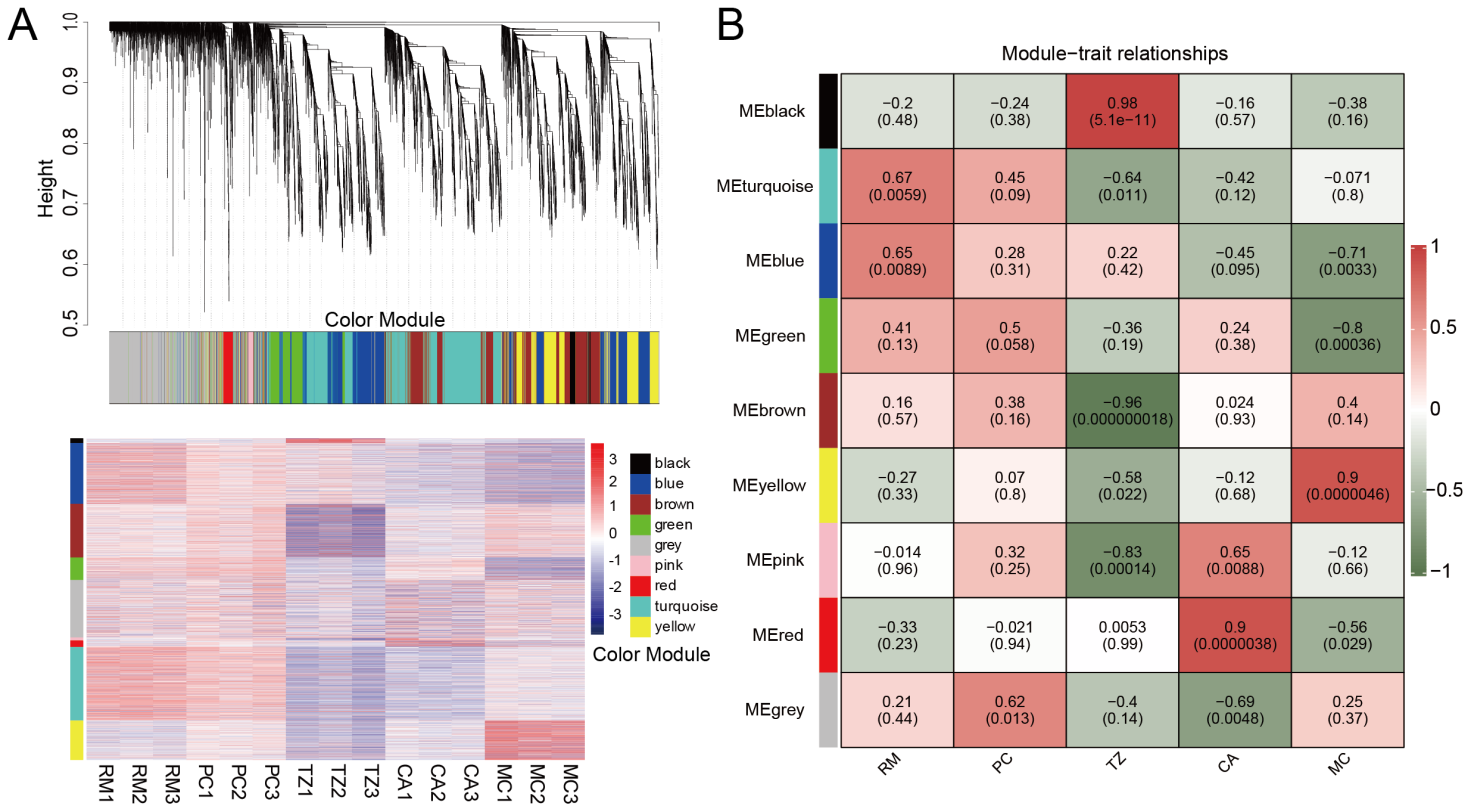
WGCNA co-expression network analysis of the antler growth center proteome. (A) Hierarchical clustering dendrogram shows co-expression modules of all identified proteins with distinct color. The module proteins are rearranged, and their expression levels are presented in the heatmap. The intensity of the color represents log2 transformed relative expression levels of each protein. (B) Proteins are assigned to nine modules. The numbers in modules represent pearson’s correlation and the numbers in parentheses represents *p* -value.

Weighted gene co-expression network analysis (WGCNA) was employed to elucidate the systemic relationships among protein groups exhibiting highly correlated co-expression patterns throughout the antler regeneration stage. The hierarchical clustering dendrogram depicted in Fig. 4A illustrated the co-expressed proteins with strong correlations, and their relative expression levels were visualized *via* a heatmap. A total of nine distinct co-expression modules were delineated with each displaying unique patterns of protein expression (Figs. 4A and 4B). The blue and turquoise module exhibited a strong correlation with the RM zone (*r* > 0.65, *p*-value < 0.05). Additionally, the black module showed a positive association with the TZ zone and a negative correlation with the Brown module. The red module demonstrated a high correlation with the CA zone, while the yellow module displayed a strong relationship with the MC zone (Fig. 4B). Following identification of the module displaying significant correlation with distinct tissue zones, GO enrichment analysis was conducted to uncover the biological processes associated with antler regeneration (Table S2). Proteins in the blue module were enriched in cellular amino acid biosynthetic process and tumor necrosis factor production, consistent with mesenchymal cell proliferation and differentiation during antler fast growth stage (Table S2). The protein expression heatmap revealed elevated expression levels of the blue module in the RM zone, with a subsequent sharp decline in the TZ zone (Fig. 4A). The proteins essential for collagen metabolomic process, including NES, CTSK, and PLOD3, were defined as hub genes in this module (Table S2). We found that the black module of hub genes mainly participated in the tricarboxylic acid cycle, which well represented the property of highly expressed proteins in TZ zone. It demonstrated that the mitochondrial microenvironment experienced a significant alteration in the transition zone (TZ) zone, which supported the enhanced energy provision necessary for the fast growth of antlers. Up-regulation of tricarboxylic acid cycle (TCA) were necessary to produce energy in support of regulation of osteoblast proliferation and osteoblast differentiation. We further hypothesize that proteins highly expressed in the MC zone and with high connectivity in the yellow module might be indispensable during the hypertrophy of chondrocytes and the mineralization of its matrix. Moreover, we found that hub genes, SMAD5, SATB2 and SPP1, in the yellow module, all of which were involved with osteogenesis. To verify this hypothesis, the mRNA expression of these genes in different tissues were tested firstly from RNA-seq result, which was found that they were all highly expressed in the cartilage and mineralized cartilage zones (Table S3). Furthermore, osteoblast proliferation related genes, ITGAV and ITGB3, were also significantly upregulated in MC zone. We also found SLC9B2 and SRC, associated with osteoclast differentiation, potentially involved extensive osteoclastic resorption of mineralized cartilage matrix. The GO result indicated that the hub genes in the yellow module regulated the mitochondrial ATP metabolic pathway, potentially meeting the energy demands of cell proliferation during endochondral ossification (Table S2).

### Comparative proteomic and transcriptomic analysis of antler growth center

**Figure 5:**
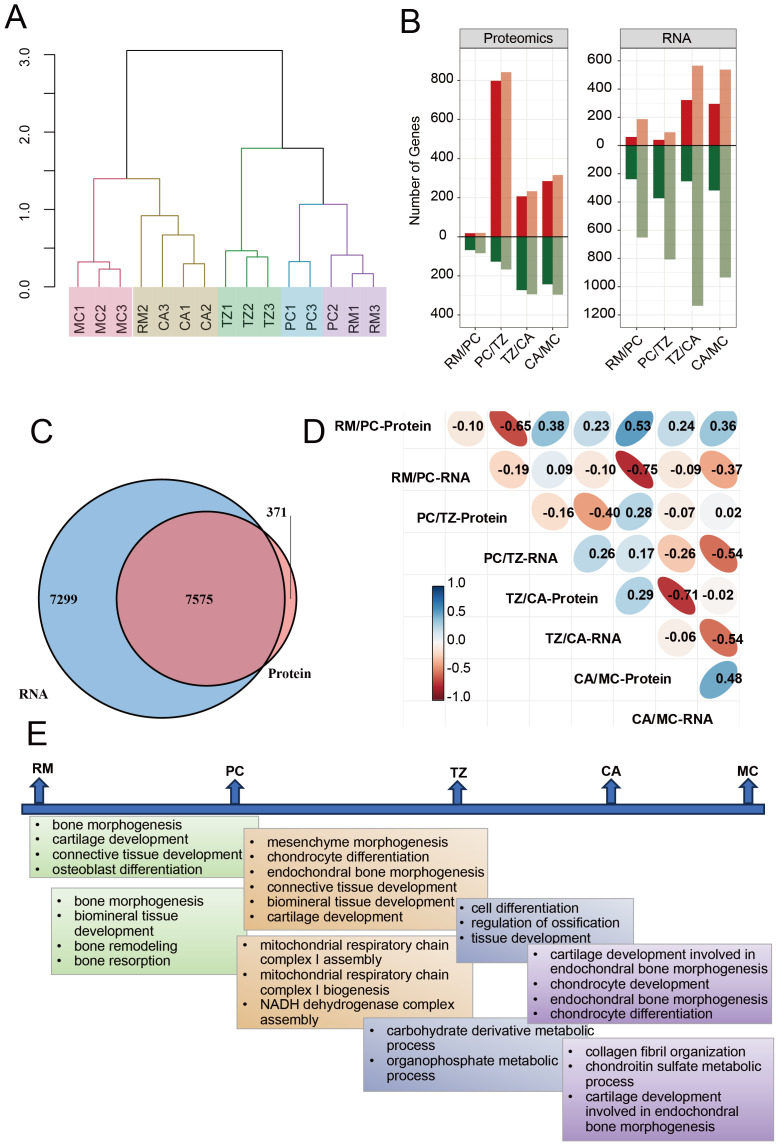
Comparative proteomic and transcriptomic analysis of the antler growth center. (A) Unsupervised clustering of mRNA in five consecutive tissue layers from RNA-seq. (B) The bar graphs of significantly different expression proteins and genes between two consecutive tissue. Light red and green represent all identified DEPs and DEGs, while dark red and green represent overlapped mRNAs and proteins. (C) Overlap of identified mRNA and proteins. (D) correlation of RNA and protein expression between two consecutive tissue layers. Pearson’s correalation coefficients displayed by shape and color. (E) GO terms of DEPs and DEGs between two consecutive tissue layers.

To comprehensively explore the correlations between RNA and protein expression level, we reanalyzed the previous published RNA-seq data (Ba et al., 2019) from each of the five consecutive tissue zones from antler tips. Hierarchical clustering and PCA results (Fig. 5A and Fig. S2) of the RNA-seq data showed a significant difference from that of the proteomic data (Fig. 1E and Fig. S2). Overall, 7,575 genes overlapped between the detected transcriptome (14,874 RNAs FPKM > 0.1 in at least one sample for each stage, Table S3) and the proteome (8,173 identified proteins, including isoforms expressed by 7,946 genes) in the whole process (Fig. 5C). We then identified the up- and down-regulated mRNA and protein between each of two consecutive stages. Transition from the RM to the CA zone resulted in more than eight hundred of up-regulated proteins (Fig. 5B). However, massive downregulation at the mRNA level was observed at the PC zone (Fig. 5B). Among the analyzed genes, the correlation between mRNA and protein expression was weaker when comparing each pair of consecutive stages, whereas the correlation in protein expression alone was stronger across stages (Fig. 5D). These findings indicated that alterations in protein expression were more gradual, while mRNA expression levels exhibited fluctuations at specific stages. To elucidate the interplay between mRNA and protein levels, we categorized the differentially expressed genes (comparing mRNA and protein expression across two successive stages) into Gene Ontology (GO) terms (Fig. 5E, Table S4). Initially, focusing on the RNA levels, we identified that terms such as regulation of developmental processes, tissue development, and osteoblast differentiation were significantly enriched when comparing the RM and PC zones (Fig. 5E). Concurrently, proteomic analysis corroborated these findings, revealing that biomineral tissue development was a predominant pathway in the chondrogenic differentiation process from the RM zone to the pre-cartilage zone. Although the protein abundance and mRNA expression level were not concordant, the biological process of significant change mRNA and protein between RM and PC mainly played important roles in bone morphogenesis. Further analysis of the changes between the PC and transitional zones (TZ) revealed that differentially expressed genes were primarily enriched in the regulation of cartilage development, including endochondral bone morphogenesis. In contrast, differentially expressed proteins (DEPs) between the PC and TZ zones were mainly associated with the mitochondrial respiratory chain. Furthermore, the DEPs between TZ and CA mainly involved in carbohydrate derivative metabolic and organophosphate metabolomic process. Additionally, the differences between CA and MC primarily encompassed cartilage development within the endochondral bone morphogenesis pathway, irrespective of whether genes or proteins were considered. The consecutive stages analysis suggested that substantial variations in protein and RNA expression may occur during the transition from the pre-cartilage to cartilage zone.

### Monotonically expressed genes and proteins identification across the antler tips

**Figure 6:**
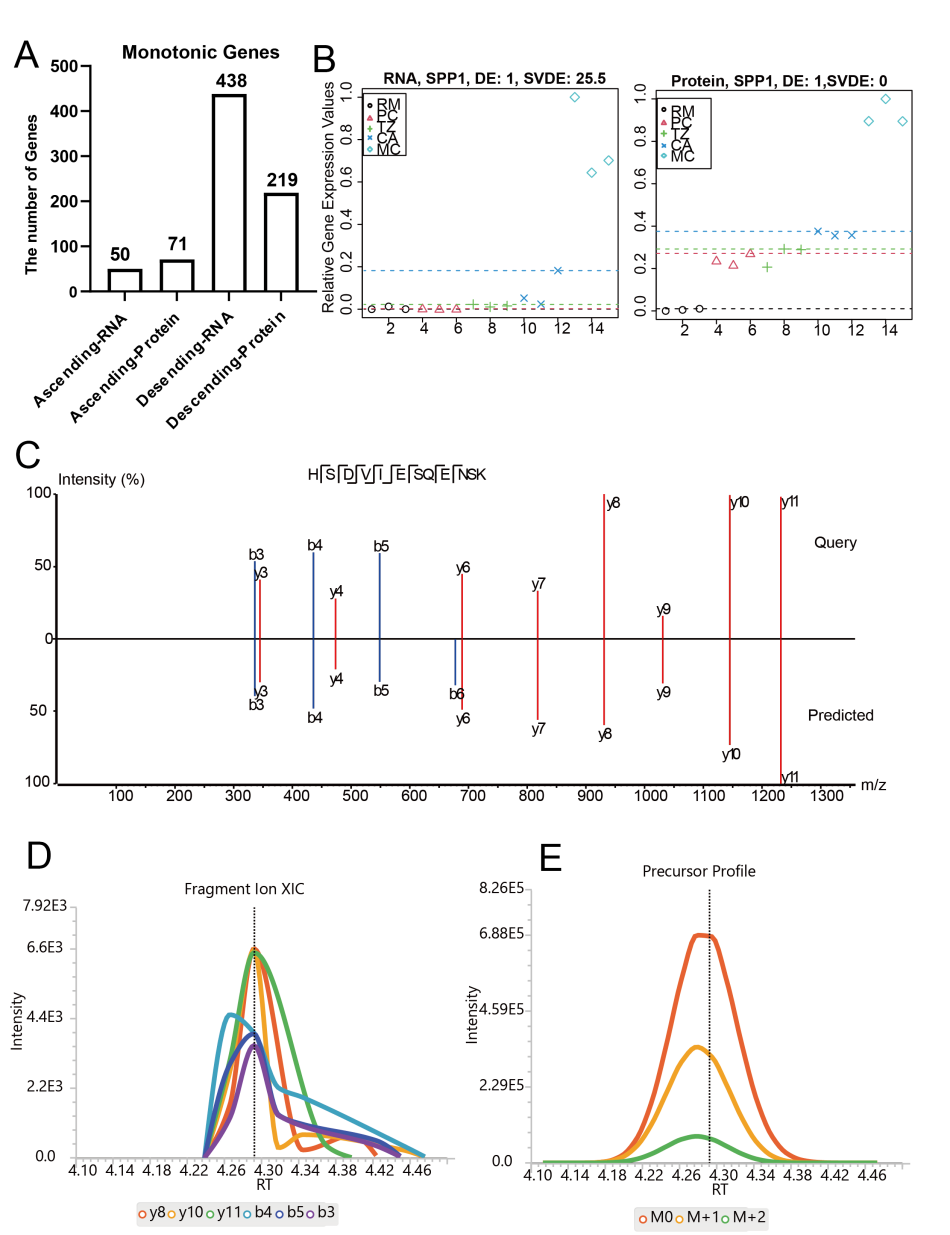
Monotonically expressed genes and proteins across the antler growth center. (A) The summary of monotonic genes and proteins. (B) Overlap monotonic marker SPP1 between RNA-seq and proteomics of discriminating lines and discriminating errors (DE) in each tissue zone. (C) MS2 spectrum of unique peptide from SPP1. (D) and (E) Extract ion chromatography (XIC) of fragment and precursor ion of SPP1 unique peptide, respectively.

To ascertain whether there were monotonic changes in gene or protein expression across five tissue zones, we employed the monotonic feature selector method to pin-point genes or proteins exhibiting robust monotonic characteristics. Our analysis revealed 438 genes and 219 proteins with monotonically decreasing expression, along with 50 genes and 71 proteins with monotonically increasing expression (Fig. 6A, Table S5). Notably, both the monotonically increasing genes and proteins were implicated in the positive regulation of hormone secretion pathways. SPP1, emerged as the only one overlap hub gene, displayed a monotonically increasing pattern in both protein and gene expression (Fig. 6B). To further verify the ascending marker SPP1, we checked the MS2 spectrum of the unique peptide (HSDVIESQENSK) that fully matched with predicted theoretical spectrum (Fig. 6C). Moreover, the extract ion chromatography (XIC) of major fragment ions could align well for quantification (Figs. 6D and 6E). SPP1, as osteoblast marker genes, was mainly involved in the attachment of osteoclasts to the mineralized bone matrix. These findings strongly indicated that SPP1 may function as a pivotal, monotonically ascending hub gene involved in the mineralization process of cartilage through the endochondral ossification mechanism. Monotonic genes that exhibited a descending pattern were predominantly implicated in cellular stress responses, the regulation of catabolic processes, RNA localization, and the biogenesis of ribonucleoprotein complexes (Table S6). Additionally, descending monotonic proteins involved in extracellular matrix organization, extracellular structure organization, connective tissue development, cartilage development and collagen fibril organization (Table S6). The relationship between the expression patterns of monotonically expressed RNA and proteins was observed to be weakly correlated, similar to the correlation analysis of differentially expressed RNA and proteins in consecutive tissues.

## Discussion

Deer antlers represent a unique model of complete organ regeneration in mammals characterized by an unparalleled rate of bone growth (Feleke et al., 2021; Wang & Landete-Castillejos, 2023). This process is underpinned by a complex interplay of cellular proliferation, differentiation, and tissue remodeling that is spatially and temporally orchestrated. Our study employed a proteomics approach to spatially resolve protein expression across five distinct zones of the growing antler tip, revealing dynamic molecular signatures that govern this rapid osteogenesis. The clustering analysis successfully grouped proteins into distinct expression patterns, providing significant insights into the functional specialization of each tissue zone, from the progenitor-rich reserve mesenchyme to the terminally differentiated mineralized cartilage.

### The reserve mesenchyme as a chondro-progenitor niche

The RM zone as a dynamic hub of progenitor cells poised for chondrogenesis was a key finding of this study. Among 10 clusters, Cluster 2, 7, and 9, which showed the highest expression level in the RM and PC zones, were enriched with critical proteins for mesenchymal cell proliferation and differentiation. PRRX1 and LUM levels were highly expressed in the RM, consistent with their role in driving mesenchymal cell condensation during differentiation (Hu et al., 2024). These findings supported the regenerative model where *PRRX1+* cells gave rise to the antler blastema. Furthermore, the co-expression of IHH, SOX9, and POSTN in this zone underscored a potent signaling environment that promoted the expansion of chondro/osteo progenitor zone. Interestingly, our data indicated that the RM zone possessed strong chondral-progenitor characteristics. Chondrocyte differentiation regulators, including POSTN, COL3A1, FGFR3, and RUNX3, were highly expressed in the outermost zone, suggesting early commitment to the cartilage lineage. Expression then decreased as cells differentiated inward. The elevated expression of SOX6 and the cartilage markers SOX9 and CHAD in the RM further solidified this finding, suggesting that the commitment of mesenchymal cells to a chondrogenic fate was an early event in antler growth. This finding, however, presented a nuanced picture when compared with recent single-cell transcriptomic studies, which reported SOX6 to be predominantly expressed in the cartilage zone (Qin et al., 2023). This discrepancy could reflect the complex regulatory landscape where mRNA and protein levels were not always directly correlated, highlighting the importance of multi-omics approaches for a complete understanding of gene regulation.

### Stem cell regulation and cancer-analogous growth during antler development

The rapid regeneration of antlers was fundamentally a stem cell-driven phenomenon (Liu et al., 2023; Zhang et al., 2021b). Our data revealed a sophisticated regulation of both ESC and MSC markers. Four ESC markers were found in cluster 5, with two expression peaks in the RM and MC zones. Notably, the transcription factor ZNF281 inhibited osteochondrogenic differentiation and stimulated MSC proliferation (Seo et al., 2013). High levels in the RM likely promoted MSC proliferation and maintained their undifferentiated state. Its expression then dropped in the TZ, permitting the initiation of chondrogenic differentiation, before rising again in the MC zone. This re-upregulation in the mineralized zone, in conjunction with HDAC4, may be a mechanism to compensate for chondrocyte hypertrophy and drive the final stages of endochondral bone formation (Vega et al., 2004). MSC marker genes predominantly grouped into Clusters 1 and 4. This distribution aligned with the findings from the antler hub gene cluster analysis, suggesting that SPP1 (Nagata et al., 1991), VCAM1 (Feuerbach & Feyen, 1997), ANPEP (Nefla et al., 2015) participated in the matrix mineralization of antler cartilage zone. Notably, SPP1 exhibited a monotonic increase in both RNA and protein levels, suggesting its role as a key regulator of endochondral differentiation.

One of the most striking aspects of antler growth is its deployment of cancer-analogous proliferative mechanisms in a controlled, non-pathological context (Li et al., 2023). Especially, the highly proliferative cells were predominantly located in the inner zone of the RM, exhibiting gene expression profiles more conducive to osteosarcoma than to normal bone tissues. Our findings lend proteomic support to this concept. The high expression of S100A4 (Wang et al., 2017) in the RM and PC zones, a protein known to promote MSC proliferation and cancer cell motility, contributed to the rapid expansion of the progenitor pool. Furthermore, the upregulation of proteins associated with cancer cell migration and invasion, such as COL1A1 (Li et al., 2022) and SCAMP1 (Yang et al., 2013), in the MC zone suggested that mechanisms typically associated with metastasis were repurposed for tissue remodeling and vascular invasion during late stage of endochondral ossification. This highlighted the antler as a unique system where cancer-analogous proliferative and invasive properties were harnessed for constructive, regenerative purposes without leading to malignant tumors.

### Spatial control of mineralization and vascularization

Comprehensive proteomic analysis provided a detailed molecular map of endochondral ossification as it unfolds across the antler tip. The process appeared to be initiated in the TZ zone, as evidenced by the peak expression of proteins in Cluster 8. The upregulation of COL10A1, a marker for hypertrophic chondrocytes, and MMP9 (Wang et al., 2013), which was involved in matrix remodeling, pinpointed the TZ as the site where chondrocytes begin to mature and prepare the cartilage matrix for mineralization. The presence of the bone mineral metabolism marker SPARC (Stéger et al., 2010) in this cluster further supported the TZ as the frontier of mineralization.

As we moved inwards to the CA and MC zones, Cluster 4 proteins became dominant. This cluster included enzymes like MMP13 (Wang et al., 2013) and CTSK (Debnath et al., 2018), which were essential for degrading the cartilage matrix to allow for vascular invasion and bone deposition. Concurrently, osteoblast differentiation markers PHEX (Alos & Ecarot, 2005) and SPP1 were upregulated, which facilitated the deposition and organization of bone matrix proteins, leading to the hardening of the tissue. This coordinated sequence of matrix degradation and synthesis was a hallmark of this modified endochondral ossification in antler tips.

Vascular development was intrinsically linked to this process. The high expression of endothelial markers CDH5 and EMCN in the TZ suggested that this zone was a hotbed of angiogenesis. This localized vascular growth was critical for transporting nutrients, oxygen, and progenitor cells to the rapidly expanding tissue and is a prerequisite for replacing the avascular cartilage with vascularized bone (Clark et al., 2006). Surprisingly, we found high levels of the endothelial marker VWF in the MC zone. While VWF was typically associated with blood vessels, its upregulation in the mineralized zone may be a response to the local hypoxic microenvironment, which was in a condition commonly observed in rapidly growing tissues and tumors (Randi, Smith & Castaman, 2018). This hypoxia, particularly in the RM and PC zones, may be a key driver of the antler’s regenerative capacity, as it was able to maintain stem cell pluripotency and promote proliferation while favoring chondrogenic over osteogenic differentiation (Na et al., 2024).

## Limitations and future study

The deep proteomic approach confidently identified low-abundance regulatory proteins such as transcription factors SOX9, SOX6, RUNX3, and ZNF281 through MS2-level verification of unique peptide fragmentation spectra. However, independent protein-level validation remains a limitation due to the scarcity of commercially validated antibodies for sika deer (*Cervus nippon*) tissue. Future studies should develop targeted proteomics approaches, particularly parallel reaction monitoring or multiple reaction monitoring, which can provide highly sensitive and quantitatively precise validation of specific proteins across the five tissue zones without relying on species-specific antibodies. To confirm zone-specific expression patterns in the future, spatial validation through immunohistochemistry will be essential, along with the development of the corresponding commercial antibody for deer.

## Conclusion

The study introduced the initial spatially resolved proteomic atlas of the deer antler growth center spanning all five consecutive tissue zones. This established a comprehensive molecular framework for endochondral ossification in a regenerating mammalian organ. The reserve mesenchyme, which was recognized as a progenitor niche, displayed co-expression of embryonic and mesenchymal stem cell markers, suggesting that antler stem cells were a special type of MSC that possess partial ESC features. This provided a proteomic foundation for the remarkable regenerative capacity of antlers. The transition zone was recognized as the crucial regulatory layer where chondrocyte hypertrophy, matrix remodeling, and vascular invasion intersect, orchestrated by spatially restricted expression of chondrocyte and endothelial markers. The significant disparity between protein and mRNA levels across tissue zones underscored the importance of direct protein measurement in developmental biology, as transcriptomic data alone are insufficient to fully depict the functional molecular landscape. The extensive proteome coverage obtained in this study, facilitated by the Orbitrap Astral platform, enabled the detection of low-abundance transcription factors at their anticipated spatial positions, confirming their involvement in chondrogenesis at the protein level. These findings offer novel mechanistic insights into bone development and serve as a valuable resource for identifying therapeutic targets in bone repair and regenerative medicine.

##  Supplemental Information

10.7717/peerj.21568/supp-1 Supplemental Information 1 Pearson’s Correlation of Protein Expression of 15 antler samples including 5 consecutive tissue groups

10.7717/peerj.21568/supp-2 Supplemental Information 2 PCA analysis of 5 consecutive tissue groups for RNA-seq data

10.7717/peerj.21568/supp-3 Supplemental Information 3 Protein identification and expression matrices from quantitative mass spectrometry

10.7717/peerj.21568/supp-4 Supplemental Information 4 GO biological process categories for significantly positive module correlated with the corresponding tissue for WGCNA

10.7717/peerj.21568/supp-5 Supplemental Information 5 mRNA expression matrices for five tissue

10.7717/peerj.21568/supp-6 Supplemental Information 6 Significant GO terms for differential expression proteins (DEPs) and genes (DEGs)

10.7717/peerj.21568/supp-7 Supplemental Information 7 The list of monotonic marker genes and proteins from RNA-seq and Proteomics

10.7717/peerj.21568/supp-8 Supplemental Information 8 GO Biological Process of Monotonic Genes and Proteins

## Additional Information and Declarations

### Data Availability

The following information was supplied regarding data availability: The data is available at ProteomeXchange Consortium: PXD066352
