# Supplementary figures and images for: Proteomic dynamics in endochondral ossification: insights from antler tip analysis
Source: PeerJ. 2026 Jul 27;14:e21568. doi: 10.7717/peerj.21568 (PMC13421809; doi:10.7717/peerj.21568)

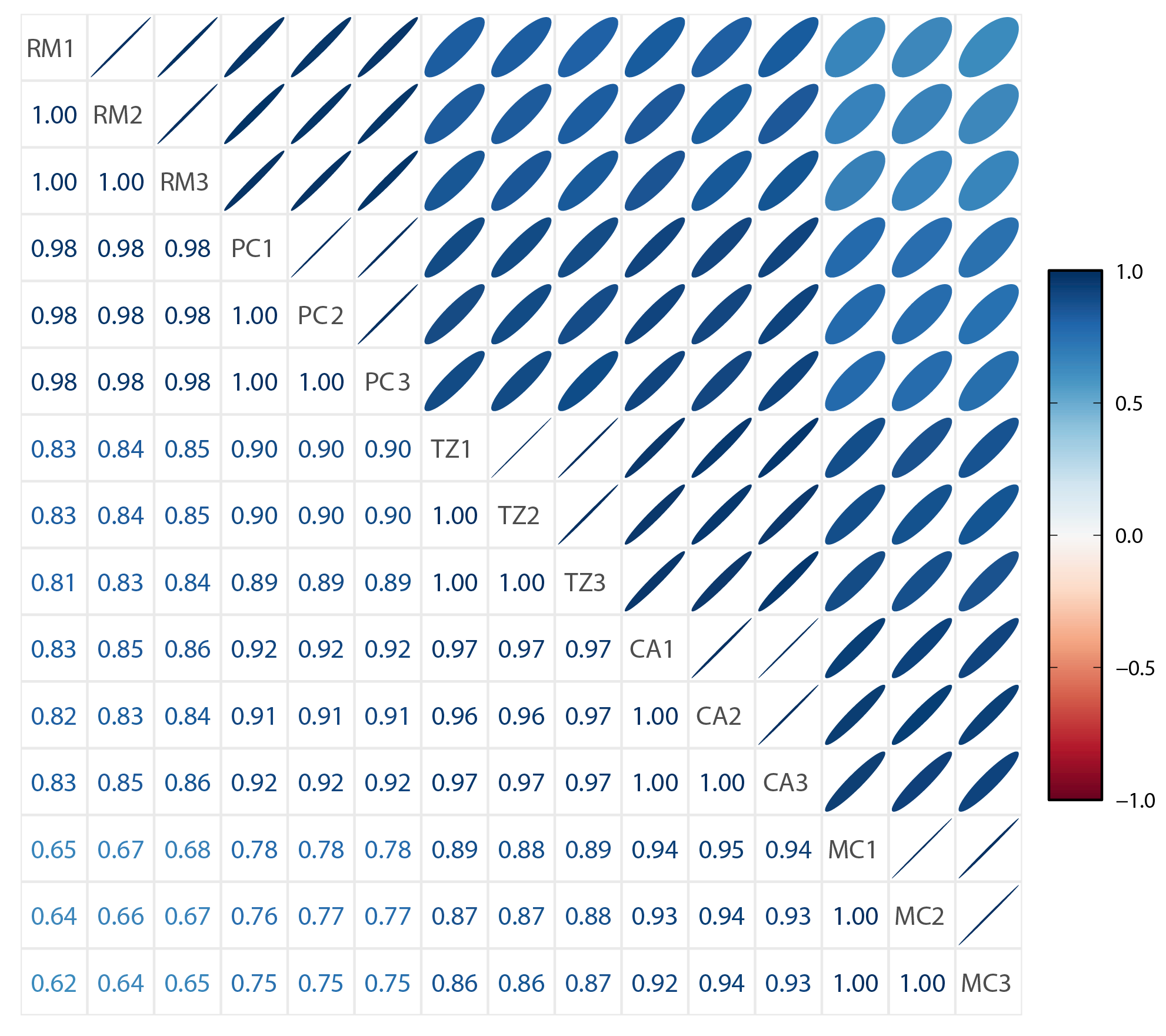

Supplement: Supplemental Information 1 [file peerj-14-21568-s001.png]

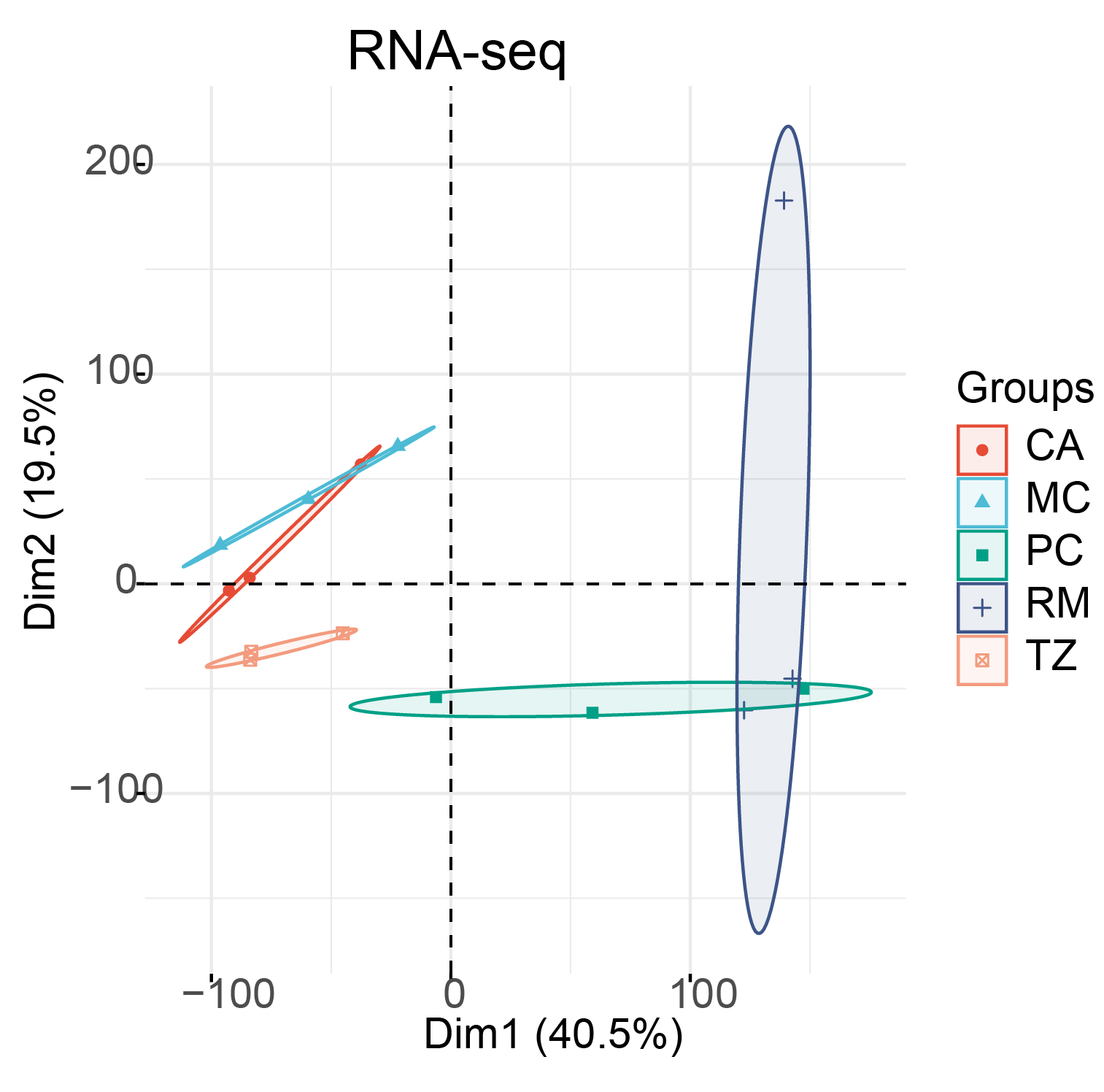

Supplement: Supplemental Information 2 [file peerj-14-21568-s002.png]
